# Safety and efficacy of antipsychotic drugs for the behavioral and psychological symptoms of dementia

**Published:** 2009-01

**Authors:** Chittaranjan Andrade, Rajiv Radhakrishnan

**Affiliations:** Department of Psychopharmacology, National Institute of Mental Health and Neurosciences, Bangalore 560 029, India

**Keywords:** Antipsychotics, dementia, behavioral and psychological symptoms of dementia, mortality, stroke

## Abstract

**Background:**

Antipsychotic drugs are commonly used in the treatment of the behavioral and psychological symptoms of dementia (BPSD).

**Materials and Methods:**

We present a qualitative review of the data on the efficacy and safety of antipsychotic drugs for BPSD. We more specifically examine safety issues with an especial focus on recent research. We examine two safety studies in detail to provide readers with a critical perspective.

**Results:**

Typical and atypical antipsychotic drugs both attenuate the severity of BPSD; however, both categories of drugs increase the risk of cerebrovascular and other adverse events, as well as the risk of death. The risk appears greater with the typical drugs, with higher doses, and during the initial weeks of treatment. The risk probably persists for as long as a year after the initiation of treatment. Both drug- and patient-related factors appear to mediate this increase in risk.

**Conclusions:**

Antipsychotic drugs should be considered for BPSD only if there is a specific need, or if other treatments have failed; decision-making should be individualized and documented after a risk-benefit analysis. Atypical antipsychotics appear safer than the typical drugs. The lowest effective dose should be used.

## INTRODUCTION

Improved healthcare has led to a higher life expectancy; as a result, the proportion of elderly persons in the population is rising. Medical and psychiatric problems associated with the elderly are consequently assuming progressively greater importance. Dementia is among the most important of these problems because it represents a chronic condition for which, so far, only palliative care is available.

The prevalence of dementia in the USA was reported at 13.9% among individuals aged 71 years and older.[1] Global estimates for 2001 showed that, worldwide, approximately 24.3 million people suffered from dementia; and 60.1% of all people with dementia lived in developing countries.[2] In India, the prevalence of dementia lies in the range of 1.8 to 3.6% among individuals aged 60 to 65 years (depending on the study) and above.[3–5]

Patients with dementia display cognitive and behavioral problems. The present article is a qualitative review which focuses on the latter, with especial reference to recent research on the subject, and with more especial reference to the risks associated with the use of antipsychotic medications in the management of the neuropsychiatric symptoms of dementia.

Behavioral and psychological symptoms of dementia (BPSD), a label coined at an international consensus conference convened by the International Psychogeriatric Association Task Force in 1996,[6] refers to disturbances such as anxiety, depression, agitation, aggression, delusions, hallucinations, inappropriate sexual behavior, wandering, insomnia, and other noncognitive symptoms that may arise in the context of a dementing illness. BPSD is common. For example, a large community-based study which screened 5092 individuals aged 65 years and older estimated that the point prevalence of any neuropsychiatric disturbance in individuals with dementia was 61%; the point prevalence for any serious disturbance was 32%.[7] A 10/66 Dementia Research Group study which evaluated subjects from 21 centers in 17 developing countries found at least one behavioral symptom to be present in 70.9% of persons with dementia.[8]

Whereas BPSD is not a separate entity with distinct nosological boundaries, it is useful as a descriptive term to outline a clinical construct that is associated with substantial patient impairment and caregiver distress; and hence a construct that merits close clinical attention.[9] In Western studies, BPSD has been identified as a significant cause for institutionalization in the elderly.[10]

## MANAGEMENT OF BPSD

Multidisciplinary measures have been proposed for the management of BPSD; these include attention to comorbid medical problems, correction of disturbances in vision and hearing, implementation of specific behavioral strategies for the reduction of identified target symptoms, and institution of measures that improve caregiver coping and quality of life.[11–13] These approaches notwithstanding, the thrust of treatment continues to be largely pharmacological because nonpharmacological strategies are difficult to implement in a clinical setting.[14] In this context, one would imagine that preference for pharmacological approaches would be even more likely in countries such as India in which the population is large, and in which the mental health manpower is not sufficiently adequate to meet the growing demand.

Pharmacological treatments that have been found effective for BPSD include cholinesterase inhibitors such as donepezil, galantamine, and rivastigmine,[15–17] the *N* -methyl-D-aspartate receptor antagonist, memantine,[18] antipsychotic drugs such as risperidone,[19] antidepressant drugs such as SSRIs[20] and trazodone,[21] mood stabilizers such as valproate[22,23] and carbamazepine,[23] and benzodiazepines such as lorazepam.[24] Antipsychotic drugs are among the most widely researched from amongst these categories, perhaps because of their broad spectrum of efficacy against the varied manifestations of BPSD.

## ARE ANTIPSYCHOTICS EFFECTIVE IN THE MANAGEMENT OF BPSD?

Typical antipsychotics are modestly effective in reducing behavioral and psychological symptoms of dementia;[25,26] as an example, low-dose haloperidol is effective and well tolerated for this indication.[27] However, the anticholinergic and extrapyramidal adverse effects of typical antipsychotic medications may preclude the use of adequately high doses, and may increase the risk of poor functional outcome and institutionalization.[28] As elderly subjects are sensitive to extrapyramidal adverse effects, atypical antipsychotic agents may appear more suitable for BPSD. In this context, a meta-analysis of different pharmacological treatments for the management of neuropsychiatric symptoms of dementia found that antidepressants, mood stabilizers, memantine, and benzodiazepines failed to show consistent evidence of benefit; the most effective (albeit only modestly so) drugs were olanzapine and risperidone.[29]

In a retrospective, naturalistic study of the effects of 6 months of treatment with risperidone, olanzapine, and quetiapine on behavioral disturbances in outpatients with mild to moderate Alzheimer’s disease, all three antipsychotics were found to significantly reduce behavioral disturbances; and all were well-tolerated with low adverse event rates. Baseline cognition scores correlated with decrease in the agitation item of the Neuropsychiatric Inventory (NPI); this suggests that neuropsychiatric symptoms may be less likely to respond to antipsychotic drugs in patients with greater baseline cognitive impairment.[19]

In a meta-analysis of 15 randomized, placebo-controlled trials of atypical antipsychotics for BPSD, Schneider *et al*.[30] found that patients treated with aripiprazole and risperidone (but not those receiving olanzapine) showed significant improvement on various rating scales; however, patients receiving antipsychotics were more likely to worsen on cognitive test scores.

The Clinical Antipsychotic Trials of Intervention Effectiveness-Alzheimer’s Disease (CATIE-AD) study specifically enrolled Alzheimer’s disease patients with associated agitation, aggression, or psychosis.[31] The study was designed to have several phases. In Phase 1, patients were randomized to receive flexible-dose treatment with olanzapine, quetiapine, risperidone, or placebo for up to 36 weeks. In subsequent phases, nonresponding patients were re-randomized in a double-blind fashion to 36 weeks of treatment with another antipsychotic (different from that which the patient had received in Phase 1) or citalopram; or patients could receive open treatment with an antipsychotic drug or citalopram. At the end of Phase1, patients had received, on average, 5.5 mg/day of olanzepine, 1 mg/day of risperidone, or 56.5 mg/day of quetiapine. Those who were randomized to olanzapine or risperidone showed significantly greater improvement on the NPI than those randomized to quetiapine or placebo; only patients randomized to risperidone showed significant improvement on the Clinical Global Impression of Change (CGI-C) measure. On the Brief Psychiatric Rating Scale (BPRS), in comparison with placebo, the hostility-suspiciousness factor improved with olanzapine and risperidone, whereas the psychosis factor improved significantly only with risperidone. There were no differences between antipsychotic drugs and placebo on measures of cognition, activities of daily living (ADL) and quality of life, except for a worsening of ADL with olanzapine.

Of note, not all antipsychotic trials have yielded favorable results. For example, in a 26-week randomized control trial, Ballard *et al*.[32] examined the safety and efficacy of quetiapine and rivastigmine in 93 patients with Alzheimer’s disease and agitation. Counterintuitively, neither quetiapine (50 mg twice daily) nor rivastigmine (9-12 mg/day) improved agitation; even more surprisingly, quetiapine-treated patients experienced significantly greater dysfunction on a severe impairment battery relative to placebo-treated patients.

## ARE ANTIPSYCHOTICS HARMFUL IN PATIENTS WITH DEMENTIA?

### Stroke

It was already apparent by 2003 that the risk of stroke was elevated in elderly patients who were receiving antipsychotic medications. For example, the pharmaceutical industry itself sounded a warning, based on the results of 4 placebo-controlled trials involving 1230 patients with dementia; in these studies, cerebrovascular adverse events were twice as common in risperidone-treated patients (4%) as in placebo-treated patients (2%) across as brief a period as 1 to 3 months.[33] The FDA subsequently released this information as a safety alert.[34] A similar industry-initiated report suggested an (nonsignificantly) increased risk of stroke associated with the use of olanzapine in elderly patients with dementia, based on 5 trials which used doses ranging from 1 to 15 mg/day across 6 to 10 weeks; the relative risk of a cerebrovascular event was 3.1 (95% CI, 0.7-13.5) and the absolute increase in risk was 0.9%.[35] Concerns about the safety of antipsychotic medications in the elderly were subsequently expressed in formal publications.[36,37] A more recent meta-analysis of 15 randomized controlled trials of atypical antipsychotics (*n* =3353) vs. placebo (*n* =1757) in patients with dementia also identified an increased risk of cerebrovascular adverse events in the atypical antipsychotic group.[30]

In a case-control study which evaluated the temporal relationship between exposure to antipsychotic drugs and cerebrovascular events, Kleijer *et al*.[38] demonstrated a robust association between treatment duration and risk, with the risk of cerebrovascular adverse events being significantly elevated with use for less than 1 week (OR, 1.7; 95% CI, 5.7-17.2) and with the risk subsequently decreasing to a nonsignificant level across a 3-month period (OR, 1.0; 95% CI, 0.7-1.3). Exposure to antipsychotic drugs (either current or recent) was associated with an overall 70% increase in the risk of cerebrovascular adverse events (OR, 1.7; 95% CI, 1.4-2.2).

The results of another study[39] also suggest that duration of exposure may influence the risk of cerebrovascular events; risk decreases with continued use. In this 18-month study which stratified patients (*n* =14,029) as either Alzheimer’s disease or vascular dementia, and which stratified antipsychotic use as either first-generation antipsychotic (FGA), second generation antipsychotic (SGA) or no antipsychotic, there was no increase in the risk of cerebrovascular adverse events among those receiving FGAs or SGAs. In a subgroup analysis, there was no increase in the risk of cerebrovascular adverse events in the Alzheimer’s subgroup, though patients with vascular dementia did suffer an increased risk.[39]

Is the risk greater with typical antipsychotics? A retrospective study of 32,710 elderly patients with dementia who were prescribed typical (*n*=14,865) or atypical (*n* =17,845) antipsychotic drugs found that the adjusted risk of ischaemic stroke was comparable in the two groups (HR, 1.01; 95% CI, 0.81-1.26).[40]

### Mortality

In April 2005, the US Food and Drug Administration (FDA) issued a public health advisory that the use of atypical antipsychotics in dementia may be associated with increased mortality.[41] This advisory was based on a review of 17 randomized, placebo-control trials of the atypical antipsychotics risperidone, olanzapine, quetiapine, and aripiprazole. In 15 of the trials, the risk ratio for atypical antipsychotics was approximately 1.6-1.7 compared to placebo. A limitation of this analysis is that the pooled mortality rate with placebo was 40 out of 1757 (2.3%) individuals; this is substantially higher than the annualized age-corrected value of 72.3/100,000 (0.07%) in patients with dementia (National Center for Health Statistics).[42] Thus, it appears that antipsychotic drugs raise the risk of morality in dementia patients who are already at high risk. Whether this high risk is a facet of the BPSD for which the atypical antipsychotics were prescribed or whether it represented selection bias (because more severely ill patients may have been recruited in clinical trials in tertiary care research centers) is unknown. We will return to this point towards the end of this article.

An examination of the use of typical antipsychotics in the elderly has led to a similar concern of increased mortality, as was seen in a retrospective cohort study involving 22,890 individuals, aged 65 years and older, who had received antipsychotic drugs. In this study, the adjusted risk of death was 37% higher (relative risk (RR), 1.37; 95% confidence interval (CI), 1.27-1.49). with typical antipsychotic drugs than with atypical antipsychotics.[43] In an earlier study, 21.4% of haloperidol-treated patients aged 65 years and older (as compared with only 4.75% of those treated with atypical antipsychotics) died during a 2-year period.[44]

In a retrospective cohort study of 27,259 dementia patients aged 66 years and older, Gill *et al*. (2007)[45] found that atypical antipsychotic use was associated with a 31-55% increased mortality rate relative to no antipsychotic use. This risk was evident as early as 30 days after the initiation of treatment; and the elevation in risk appeared to persist for as long as 180 days. Furthermore, at all time points, typical antipsychotic use was associated with a marginally higher risk of death relative to atypical antipsychotic use.

In another retrospective cohort study of 37,241 dementia patients aged 65 years and older, the 180-day adjusted all-cause mortality rate in those who received typical antipsychotics (*n* =12,882) was 32% higher than that in those who received atypical antipsychotics (*n* =24,359). Among the typical drugs, the risk was highest with haloperidol and lowest with loxapine. The risk was also greater with higher doses and during the initial 40 days of treatment.[46]

In view of these data on the risks associated with the typical antipsychotic drugs, on June 16, 2008, the FDA modified its previous safety advisory to include conventional antipsychotics, as well.[47]

Other concerns related to antipsychotic use in dementia include an increased risk of sedation, gait disturbances, orthostatic hypotension, and osteoporosis, all of which can increase the risk of falls and fractures.[48] However, a meta-analysis of 15 randomized controlled trials of atypical antipsychotic drugs vs. placebo found no elevation in the risk of syncope, falls, or injury.[30]

## RECENT STUDIES RELATED TO RISKS

There have been several other large studies which recently examined the risks associated with antipsychotic medication use in elderly subjects with dementia. Two of these studies are discussed in some detail to help the reader obtain a clearer perspective.

### Kales *et al*.(2007)[49]

These authors conducted a retrospective study in the USA of 10,615 outpatients with dementia, all of whom had been newly started on psychotropic medications. All patients were above 65 years of age. Patients who had been started on antipsychotic medications for neuropsychiatric symptoms of dementia were compared with those who had been started on other psychiatric medications. Statistical methods were used to control for various confounding variables.

The authors found that dementia patients started on typical, atypical, or both categories of antipsychotic medications had significantly higher 1-year mortality rates (23% to 29%) than those started on nonantipsychotic medications (15%). Adjusted 1-year mortality rates for atypicals and for combined typical and atypical antipsychotic drugs were similar to those for typical antipsychotic drugs. With the exception of anticonvulsant medications, the adjusted risks with all classes of nonantipsychotic drugs were significantly lower than the risk with antipsychotic drugs. The mortality rates did not change across 12 months. Finally, the proportions of patients receiving antipsychotics who died from cerebrovascular, cardiovascular, or infectious causes were not higher than those in patients taking nonantipsychotic psychiatric medications. The higher mortality rates in patients taking antipsychotic medications appeared to be due to dementia-related causes.

### Rochon *et al*.(2008)[50]

These authors described a Canadian, population-based, retrospective study which examined the risk of serious adverse events associated with antipsychotic medication use by elderly patients with dementia. Serious adverse events were defined as any events which resulted in an acute care hospital admission or death within 30 days of initiating antipsychotic therapy.

The sample comprised 20,682 elderly community-dwelling subjects and 20,559 elderly persons living in a nursing home, all of whom carried a diagnosis of dementia. The community and nursing home cohorts each comprised 3 equally-sized matched groups: those receiving an atypical antipsychotic drug, those receiving a conventional antipsychotic drug, and those receiving no antipsychotic drug. Risks were calculated after adjustment for confounding variables.

The authors found that, relative to those who received no antipsychotic therapy, the community-dwelling subjects who started atypical antipsychotic medication had a more than trebled risk (RR, 3.2; 95% CI, 2.8-3.7) of a serious adverse event during the next 30 days. Relative to those who received no antipsychotic therapy, the community-dwelling subjects who started conventional antipsychotic medication had a nearly quadrupled risk (RR, 3.8; 95% CI, 3.3-4.4) of a serious adverse event during the next 30 days. Similar but slightly less marked findings were obtained in the nursing home subjects; the relative risks were 1.9 and 2.4 for atypical and conventional antipsychotic groups, respectively.

## GENERAL CONCLUSIONS

The data that we have reviewed point to two clear conclusions:

1. In elderly patients with dementia, typical and atypical antipsychotic drugs both modestly attenuate the severity of BPSD.
2. In elderly patients with dementia, typical and atypical antipsychotic drugs both increase the risk of cerebrovascular adverse events, serious adverse events, and all-cause mortality; the risk is apparent as early as a month after the initiation of medication, probably persists for at least a year, and appears to be greater with the typical relative to the atypical drugs. Higher doses may be associated with greater risk.

## CRITICAL COMMENTS

In studies such as these, readers should be aware that no amount of matching or statistical adjustments for confounds can validate a comparison of apples and oranges; that is, patient populations which are intrinsically different by definition. Here, patients who require antipsychotics and those who do not could be intrinsically different; that is, like apples and oranges. Therefore, studies such as those of Kales *et al*.[49] and Rochon *et al*.[50] only identify significant associations; they cannot implicate antipsychotic treatment as the reason for the elevated adverse event or mortality risk. So, either or both of the following may be true: antipsychotic medications may have a direct harmful effect; or the symptoms for which the antipsychotic medications are prescribed may be associated with a worse prognosis. The findings of Kales *et al*. certainly suggest that the latter is true because the higher risk of death with antipsychotic medication appeared to be due to dementia-related causes. In this context, Scarmeas *et al*.[51] observed that psychotic symptoms were an independent predictor of mortality in Alzheimer’s patients who were followed up for up to 14 years.

The above notwithstanding, a meta-analysis of 15 randomized, placebo-controlled trials[52] found that mortality was significantly higher in patients who received atypical antipsychotics (*n* =3353) as compared with those who received placebo (*n* =1757); the figures were 3.5% vs. 2.3%, respectively (OR, 1.54; 95% CI, 1.06-2.23). As randomized controlled trials are a gold standard for the evaluation of safety and efficacy, these data establish that antipsychotic medications *per se* increase the risk. However, as we pointed out earlier in this article, this 2.3% mortality rate in the placebo group was substantially above the adjusted rate in the population; therefore, these data also establish that the indication for antipsychotic use is associated with an elevated risk.

## CONCLUDING NOTES

What should the reader receive as the take-home message concerning risks associated with antipsychotic use in patients with BPSD? It appears that the higher risk is due to *both* drug- and patient-related factors.

There are no data to suggest that the risk of serious adverse events is increased with the other treatments for BPSD, listed earlier in this article. Therefore, we suggest that antipsychotic drugs should be considered for BPSD only if there is a specific need, or if other treatments have failed. Decision-making should be individualized after a risk-benefit analysis. If considered necessary, atypical antipsychotics should be preferred as they appear safer than the typical drugs. The lowest effective dose should be used. All decision-making should involve patients and caregivers, as applicable, and should be recorded.
